# Molecular Surveillance and Evolutionary Dynamics of Porcine Circovirus Types 2 and 3 in China, 2022–2025

**DOI:** 10.1155/tbed/6670465

**Published:** 2026-06-09

**Authors:** Yanhong Chen, Yi Lu, Yang Zeng, Yaofang Hu, Ling Dong, Mingkai Lei, Panpan Li, Weidong Yan, Anan Jongkaewwattana, Wentao Li, Xuexiang Yu, Qigai He

**Affiliations:** ^1^ National Key Laboratory of Agricultural Microbiology, College of Veterinary Medicine, Huazhong Agricultural University, Wuhan, 430070, Hubei, China, hzau.edu.cn; ^2^ The Cooperative Innovation Center for Sustainable Pig Production, Huazhong Agricultural University, Wuhan, 430070, Hubei, China, hzau.edu.cn; ^3^ College of Agronomy and Biotechnology, Southwest University, Chongqing, 400715, China, swu.edu.cn; ^4^ Virology and Cell Technology Research Team, National Center for Genetic Engineering and Biotechnology (BIOTEC), National Science and Technology Development Agency (NSTDA), Pathum Thani, 12120, Thailand, nstda.or.th

**Keywords:** epidemiological investigation, phylogenetic analysis, porcine circovirus, selection pressure

## Abstract

Porcine circovirus types 2 (PCV2) and 3 (PCV3) are globally distributed pathogens associated with reproductive disorders, subclinical infections, and multifactorial disease complexes in swine. To elucidate their epidemiological and evolutionary patterns in China, we examined 8426 clinical samples collected from 28 provincial‐level regions between 2022 and 2025. PCV2 showed substantially higher prevalence than PCV3 across production stages and clinical categories and exhibited pronounced seasonal peaks in autumn. PCV2 was most frequently detected in tissue samples and presented high viral loads in systemic wasting and enteric cases, whereas PCV3 occurred at lower viral loads and was predominantly identified in swabs, semen, and milk. A total of 181 PCV2 and 106 PCV3 ORF2 sequences were obtained for genetic analysis. PCV2d was confirmed as the overwhelmingly dominant genotype nationwide, while PCV3c predominated among circulating PCV3 lineages alongside coexisting PCV3a and PCV3b clusters. Selection pressure analyses indicated that both viruses were largely shaped by purifying selection, although PCV2 harbored a greater number of positively selected sites located in surface‐exposed regions of the Cap protein. Phylodynamic inference revealed long‐term stability in the effective population size of PCV2, whereas PCV3 experienced a recent expansion followed by stabilization. Spatial phylogeographic reconstruction demonstrated a centralized diffusion pattern for PCV2, with Hebei serving as a major transmission hub, in contrast to the multicentered and geographically admixed spread of PCV3. Collectively, these findings indicate that PCV2 remains the primary pathogenic burden in Chinese swine herds. In contrast, the more diffuse dissemination of PCV3 from multiple sources warrants sustained molecular monitoring, particularly in breeding populations.

## 1. Introduction

Porcine circoviruses (PCVs) are nonenveloped, single‐stranded circular DNA viruses classified within the genus *Circovirus* of the family Circoviridae and are among the smallest known viruses infecting mammals [1]. To date, four porcine circovirus types have been identified in pigs: PCV1, PCV2, PCV3, and PCV4. In addition, a recently reported single‐stranded circular DNA virus detected in pigs in China has been tentatively designated PCV5 [2]. PCV1 was first identified in 1974 as a contaminant in PK‐15 cell cultures. Although capable of replicating in vitro, it is regarded as nonpathogenic to pigs and was subsequently isolated again in 1982 [3, 4]. In 1997, Allan et al. [5] identified a PCV‐like virus in pigs suffering from postweaning multisystemic wasting syndrome (PMWS). Subsequent characterization revealed high homology to the earlier PK‐15 contaminant, but a more detailed sequence comparison showed that the nucleotide identity was below 80%, and the novel virus was therefore designated as PCV2 [5, 6]. In 2016, a new porcine circovirus, designated PCV3, was identified in pigs with cardiac and multisystemic inflammation in the United States [7]. Subsequently, a genetically distinct circovirus was detected in pigs with severe clinical disease in China and was named PCV4 [8]. More recently, a novel single‐stranded circular DNA virus detected in pigs with respiratory, diarrheal, and reproductive failure in southern China was tentatively named porcine circovirus type 5 [2].

In contrast to the nonpathogenic nature of PCV1, PCV2 is recognized as the primary etiological agent of PCV‐associated disease (PCVAD), a disease complex encompassing PMWS, porcine respiratory disease complex (PRDC), porcine dermatitis and nephropathy syndrome (PDNS), reproductive disorders, acute pulmonary edema, and several enteric conditions [5, 9–11]. Currently, PCV2 strains are classified into nine genotypes (PCV2a–PCV2i) [12]. Retrospective analyses indicate that PCV2a predominated during the initial global outbreaks of PCVAD. Between 2003 and 2005; however, the dominant genotype shifted from PCV2a to PCV2b, as shown by retrospective molecular epidemiological studies [13–16]. A second major shift occurred around 2008 when PCV2d emerged as the predominant genotype worldwide [17, 18]. Today, PCV2 is globally widespread across commercial swine populations, underscoring the need for continued surveillance of its pathogenic potential and evolving epidemiological patterns.

PCV2 has long been regarded as the primary etiologic agent of PCVAD. PCV3 has also been detected in pigs presenting PDNS‐like lesions [7, 19]. An infectious clone of PCV3 was successfully constructed. Under experimental conditions, the inoculation of piglets was reported to induce certain clinical signs. However, the results of experimental infection are not entirely consistent with those observed in naturally occurring diseases [20]. In addition, PCV3 has been increasingly associated with reproductive disorders, supported by high detection rates in aborted or stillborn fetuses [21, 22]. Since its initial identification in the United States in 2016, PCV3 has shown relatively low genetic diversity and a slower evolutionary rate than PCV2, indicating a highly conserved genome [23]. PCV3 classification remains inconsistent across studies. Previous reports have variably recognized two major clades with intermediate lineages or alternative subtype groupings depending on the genomic region analyzed and the phylogenetic criteria applied [24, 25]. In the present study, PCV3 strains were classified using an ORF2‐based phylogenetic framework and grouped into three clusters, designated PCV3a, PCV3b, and PCV3c [26].

Since their discovery, PCV2 and PCV3 have been reported in pig‐producing regions worldwide, including Asia, Europe, and the Americas, indicating their broad geographic distribution. In Asia, widespread detection has been documented in China, Korea, and Thailand [27, 28]. In Europe, both viruses have been identified in Germany, Denmark, Italy, and Spain [29–31]. Reports from the United States also confirm their circulation in North American swine populations [32]. Collectively, these studies demonstrate that PCV2 and PCV3 have established a global presence across major pig‐producing countries.

PCV2 is considered the principal etiological agent of PCVAD. In contrast, PCV3 has been detected in pigs with various clinical conditions and is increasingly reported as a coinfecting virus in swine populations. Both viruses may contribute to immune dysregulation, predisposing pigs to secondary infections and exacerbating disease progression, although the clinical significance of PCV3 remains under investigation [19, 33]. Coinfection with PCV2 and PCV3 has been increasingly reported in many countries, emphasizing the need for comprehensive, long‐term epidemiological surveillance across multiple regions and sample types [12, 34, 35]. These findings underscore the necessity for long‐term, multiregional, and multisample‐type systematic epidemiological surveillance to more accurately characterize viral transmission dynamics and inform prevention and control strategies.

Over the past decade, PCV2 vaccination has been widely implemented in Chinese swine herds, primarily using commercially available PCV2a‐based inactivated or subunit vaccines, sometimes in combination with other pathogens such as PRRSV. These vaccines have substantially reduced the clinical burden of PCVAD but provide only partial cross‐protection against heterologous PCV2d strains, which have become predominant in China and many other regions worldwide in recent years [36–38]. During the 2022–2025 study period, PCV2 vaccination was routinely applied in most large‐scale farms as part of standard health management programs, whereas no licensed PCV3‐specific vaccines were available. This evolving vaccine‐genotype mismatch context is important for interpreting the evolutionary patterns and putative immune‐driven selection observed in the present study.

From January 2022 to June 2025, a total of 8426 samples were collected from 28 provincial‐level regions across China, including 5046 samples from 1653 pig farms tested for PCV2 and 3380 samples from 1394 farms tested for PCV3. This study aimed to elucidate the infection status and geographic distribution of PCV2 and PCV3 in China and to conduct molecular epidemiological and phylogenetic analyses based on ORF2 gene sequences. By integrating multiyear, multiregional, and multisample datasets, our findings provide updated insights to support the epidemiological assessment, vaccine strategy optimization, and clinical diagnosis of PCVADs.

## 2. Materials and Methods

### 2.1. Collection and Processing of Clinical Samples

Clinical samples were obtained from the Center for Animal Disease Diagnosis, Huazhong Agricultural University. From January 2022 to June 2025, a total of 8426 samples were collected from 28 provincial‐level regions across China, including 5046 samples from 1653 pig farms tested for PCV2 and 3380 samples from 1394 farms tested for PCV3. Samples were collected from pigs at different production stages, including aborted fetuses, suckling piglets, nursery pigs, finishing pigs, sows, and boars. Specimen types included whole blood, anticoagulated blood, serum, oral swabs, nasal swabs, fecal swabs, environmental swabs, boar semen, sow milk, and tissue samples. Tissue specimens comprised the heart, liver, spleen, lung, kidney, umbilical cord, placenta, brain, intestinal tissues, and lymph nodes. In this study, most samples were tested for a single pathogen (PCV2 or PCV3) according to the diagnostic purpose, and all samples were tested individually without pooling. For analyses related to clinical manifestations, a subset of 1601 tissue samples with complete clinical background information was simultaneously tested for both PCV2 and PCV3. These 1601 tissue samples were categorized into six clinical groups: systemic wasting, enteric diseases, reproductive failure, neuromuscular disorders, acute death, and subclinical animals with poor performance but no obvious clinical signs.

Collected tissue samples were trimmed and transferred into sterile 2 mL centrifuge tubes, followed by the addition of 1 mL of DMEM. After homogenization using a tissue grinder (60 Hz, 360 s), the samples were subjected to a freeze–thaw cycle at −80°C, which was repeated twice. The homogenates were then centrifuged at 12,000 × *g* for 10 min, and the resulting supernatants were collected. Serum, whole blood, and anticoagulated blood samples were directly centrifuged at 12,000 × *g* for 10 min. Oral swabs, nasal swabs, fecal swabs, and environmental swabs were placed into centrifuge tubes containing 1 mL of DMEM, vortexed thoroughly, and then centrifuged at 12,000 × *g* for 10 min. Boar semen samples were thoroughly mixed and centrifuged at 12,000 × *g* for 10 min. Sow milk samples were thoroughly mixed and centrifuged at 12,000 × *g* for 10 min; the upper fat layer was discarded, and the middle aqueous layer was collected. Supernatants from all processed samples were then used for subsequent nucleic acid extraction and qPCR detection.

### 2.2. Detection of PCV2/PCV3 in Clinical Samples

Viral nucleic acids were extracted using the Vazyme DNA/RNA Extraction Kit (RM‐201‐02, Vazyme, China) following the manufacturer’s protocol. The eluates were resuspended in 50 μL of nuclease‐free water and stored at −80°C until use. Real‐time PCR assays targeting PCV2 and PCV3 were performed using primers and probes, as listed in Table 1. Each 20 μL reaction contained 10 μL of 2 × AceQ Universal U + Probe Master Mix (Vazyme, China), 0.4 μL of each primer, 0.24 μL of the probe, and 3 μL of template DNA, and nuclease‐free water. The cycling conditions were 37°C for 2 min and 95°C for 30 s, followed by 39 cycles of 95°C for 15 s and 60°C for 1 min. Conventional PCR amplification of the ORF2 genes of PCV2 and PCV3 was carried out using ORF2‐specific primer pairs (Table 1). Each 20 μL reaction consisted of 10 μL of 2 × Es Taq Master Mix, 1 μL of each primer, 3 μL of DNA, and 5 μL of nuclease‐free water. Thermocycling parameters were 94°C for 5 min, 40 cycles of 94°C for 45 s, 50°C for 45 s, and 72°C for 55 s, followed by 72°C for 10 min. PCR products were resolved on 1% agarose gels stained with GoldenView dye (Aidlab, China) and electrophoresed at 150 mA and 150 V for 15 min. Amplicons with expected band sizes were submitted to Sangon Biotech (China) for Sanger sequencing to obtain complete ORF2 sequences.

**Table 1:** The primer sequences used in this study.

Primer	Sequence (5′–3′)	Length (bp)
PCV2‐qF	GGAGTCTGGTGACCGTTGC	107
PCV2‐qR	CCAATCACGCTTCTGCATTTT	
PCV2‐qP	FAM‐CCGCTCACTTTCAAAAGTTCAGCCA‐BHQ1	
PCV2‐ORF2F	GCGATTTTGGAAGAATGCTAC	954
PCV2‐ORF2R	ACAGGTCCGCTTCTTCCATTC	
PCV3‐qF	TATTCATTAGGAGGCCCACA	142
PCV3‐qR	GCAGTTTCCCATTCGTTTAG	
PCV3‐qP	FAM‐ACTCCACCATGAACGTCATTTCC‐TAMRA	
PCV3‐ORF2F	TTACTTAGAGAACGGACTTGTAACG	649
PCV3‐ORF2R	AAATGAGACACAGAGCTATATTCAG	

### 2.3. Statistical Analysis

Statistical analyses were performed using GraphPad Prism 9.5 (GraphPad Software, USA), SPSS 26.0 (IBM, USA), and R version 4.2.2. Positivity rates and their 95% confidence intervals (CIs) were calculated using the Wilson method. Differences in detection rates among regions, seasons, sample types, and production stages were evaluated using the chi‐square test, with Fisher’s exact test applied when any expected count fell below five. For seasonal analyses, months were grouped into spring (March–May), summer (June–August), autumn (September–November), and winter (December–February). Viral load data (copy numbers) were examined for normality using the Shapiro–Wilk test. Because the data did not follow a normal distribution, comparisons among multiple clinical categories were conducted using the Kruskal–Wallis test, followed by the Mann–Whitney *U* test for pairwise comparisons. Ct values were used only for the descriptive stratification of viral load levels. Statistical significance was defined as *p* < 0.05 (two‐tailed). Spatial distribution and temporal patterns of infection were visualized using ArcGIS Pro (Esri, USA) and R.

### 2.4. Phylogenetic Analysis of PCV2/PCV3 ORF2 Genes

A total of 32 reference ORF2 sequences of PCV2 and 30 reference ORF2 sequences of PCV3 were retrieved from GenBank. For PCV2, reference sequences were selected according to the standardized ORF2 genotyping framework proposed by Franzo et al. [36], covering representative strains of the major genotypes (Supporting Information 1: Table S1), and genotype assignment was performed based on phylogenetic clustering under this framework. For PCV3, reference sequences were used to support ORF2‐based phylogenetic reconstruction and cluster assignment (Supporting Information 1: Table S2). Given the lack of a universally accepted PCV3 classification framework, strains in this study were assigned to three ORF2‐based phylogenetic clusters, designated PCV3a, PCV3b, and PCV3c, with reference to previously reported clustering patterns [26]. Before phylogenetic and selection analyses, recombination was assessed using RDP5. Sequences showing strong evidence of recombination (*p*  < 0.05 supported by ≥2 methods) were excluded from downstream analyses. These sequences, together with the ORF2 sequences obtained in this study, were aligned using MAFFT v7.505 with the “auto” strategy. To improve alignment quality, poorly aligned or ambiguously homologous regions were removed using Gblocks under a less‐stringent selection mode. Maximum‐likelihood (ML) phylogenetic trees were inferred using IQ‐TREE v2.2, with the best‐fit substitution model automatically selected by ModelFinder. Branch support was assessed using 1000 ultrafast bootstrap replicates. The resulting phylogenetic trees were visualized and annotated using the ChiPlot online platform to illustrate genotype clustering and relationships among different viral sequences.

### 2.5. Selection Pressure Analysis of PCV2/PCV3 ORF2 Genes

To identify positively selected amino acid sites within the ORF2 genes of PCV2 and PCV3, nucleotide sequences were aligned using MAFFT, and the resulting alignments were converted into codon‐based alignments in MEGA. ML phylogenetic trees were then constructed using IQ‐TREE and used as the evolutionary framework for subsequent selection analyses. Selection pressure analyses were carried out in Hypothesis Testing Using Phylogenies (HyPhy) using four codon‐based methods: Single‐Likelihood Ancestor Counting (SLAC), Fixed Effects Likelihood (FEL), Mixed Effects Model of Evolution (MEME), and Fast Unconstrained Bayesian Approximation (FUBAR). A significance threshold of *p* < 0.1 was applied for SLAC, FEL, and MEME, whereas FUBAR results were considered significant when the posterior probability exceeded 0.9. To visualize the structural distribution of positively selected sites, a reference experimental structure of the PCV2 Cap protein (PDB: 3JC1) [39] was selected based on SWISS‐MODEL template search results, taking into account the template origin, sequence identity, and oligomeric assembly state, and the selected sites were mapped onto both the monomeric structure and the 60‐mer capsid assembly. Because no high‐resolution public atomic coordinates suitable for residue‐level mapping are currently available for PCV3, PCV3 Cap monomer structures were predicted using ColabFold, whereas a homology‐based 60‐mer assembly model was generated using SWISS‐MODEL. Given the low prediction confidence of the N‐terminal region in the full‐length PCV3 Cap protein, an N‐terminally truncated model lacking the first 32 amino acids was further constructed for structural visualization. The resulting models and templates were processed and visualized in PyMOL (v2.6), where residues under positive selection were highlighted using distinct colors.

### 2.6. Phylogenetic and Phylodynamic Analysis of PCV2/PCV3 ORF2 Genes

To more comprehensively reflect the long‐term evolutionary backgrounds of PCV2 and PCV3, two levels of datasets were constructed for different analytical purposes in this study. For time‐scaled phylogenetic analysis, evolutionary rate estimation, and reconstruction of effective population size dynamics, international reference sequences from multiple countries with clear sampling time information were incorporated in addition to the sequences generated in this study, and global ORF2 sequence datasets were therefore constructed for PCV2 and PCV3, respectively. The global datasets used for time‐scaled phylogenetic and phylodynamic analyses comprised 259 PCV2 ORF2 sequences (Supporting Information 2: Table S3) and 147 PCV3 ORF2 sequences (Supporting Information 3: Table S4), including sequences obtained in this study and representative international reference sequences. For discrete‐trait phylogeographic reconstruction at the provincial level within China, province‐level datasets were constructed using only the sequences generated in this study with unambiguous province‐of‐origin information in order to analyze the transmission structures and diffusion routes of PCV2 and PCV3 among different provinces in China. Time‐calibrated phylogenies were inferred with BEAST v1.10.4 under the GTR + Γ substitution model and an uncorrelated lognormal relaxed molecular clock. A Bayesian Skygrid coalescent prior was used to infer temporal changes in the effective population size (Ne). Ancestral geographic reconstruction and cross‐regional diffusion were performed within a discrete trait framework with Bayesian stochastic search variable selection (BSSVS) enabled to identify statistically supported migration pathways. MCMC chains were run for 2 billion generations, with sampling every 20,000 steps. After removing the initial 10% of samples as burn‐in, convergence and effective sample sizes were assessed in Tracer v1.7, and all parameters showed an ESS > 200. Maximum clade credibility (MCC) trees were generated using TreeAnnotator and visualized in FigTree. Bayes factors were computed to evaluate the statistical support for individual migration routes. Major diffusion pathways were subsequently mapped onto geographic layers using ArcGIS Pro, yielding a spatiotemporal transmission network of PCV2 and PCV3 across Chinese pig populations.

## 3. Results

### 3.1. Prevalence and Geographic Distribution of PCV2 and PCV3

From January 2022 to June 2025, a total of 8426 clinical samples were collected. The farm‐level prevalence of PCV2 was 47.13% (779/1653; 95% CI: 44.73%−49.54%), and the sample‐level prevalence was 28.58% (1442/5046; 95% CI: 27.35%−29.84%). In contrast, PCV3 showed a farm‐level prevalence of 14.63% (204/1394; 95% CI:12.88%−16.59%) and a sample‐level prevalence of 13.96% (472/3380; 95% CI:12.84%−15.17%). These results indicate that PCV2 remains highly endemic in Chinese pig herds, whereas PCV3 exhibits substantially lower detection rates at the population level (Table 2).

**Table 2:** Prevalence of PCV2 and PCV3 at the sample and farm levels from 2022 to 2025.

Year	Prevalence at sample level		Prevalence at farm level	
	PCV2 positive rate	PCV3 positive rate	PCV2 positive rate	PCV3 positive rate
2022	52.11% (654/1255, 95% CI: 49.35%−54.86%)	18.49% (291/1574, 95%CI:16.65%−20.48%)	77.48% (344/444, 95%CI:73.37%−81.12%)	21.74% (85/391, 95% CI: 17.94%−26.09%)
2023	21.44% (466/2174, 95% CI: 19.76%−23.21%)	10.27% (100/974, 95% CI: 8.51%−12.33%)	33.59% (266/792, 95% CI: 30.38%−36.95%)	12.52% (83/663, 95% CI: 10.21%−15.26%)
2024	22.16% (220/993, 95% CI: 19.68%−24.84%)	9.82% (54/550, 95% CI: 7.60%−12.59%)	38.74% (141/364, 95% CI: 33.87%−43.83%)	10.86% (34/313, 95% CI: 7.88%−14.80%)
2025	16.35% (102/624, 95% CI: 13.65%−19.45%)	9.57% (27/282, 95% CI: 6.66%−13.57%)	52.83% (28/53, 95% CI: 39.66%−65.62%)	7.41% (2/27, 95% CI: 2.06%−23.37%)
Total	28.58% (1442/5046, 95% CI: 27.35%−29.84%)	13.96% (472/3380, 95% CI: 12.84%−15.17%)	47.13% (779/1653, 95% CI: 44.73%−49.54%)	14.63% (204/1394, 95% CI: 12.88%−16.59%)

**Figure 1:**
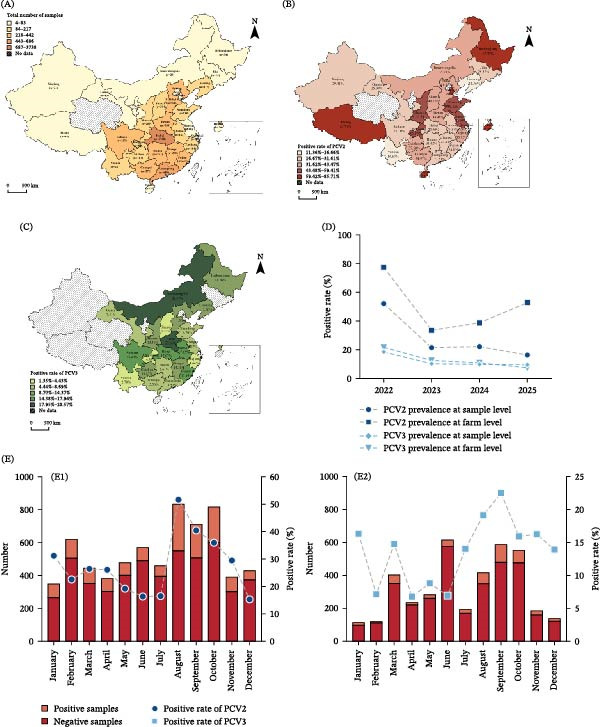
Spatial, temporal, and seasonal patterns of PCV2 and PCV3 circulation in China from 2022 to 2025. (A) Geographic distribution of the total number of samples collected across 28 provincial‐level regions. (B) Provincial‐level prevalence of PCV2, showing heterogeneous circulation intensity across China. (C) Provincial‐level prevalence of PCV3, generally lower than that of PCV2 but displaying distinct regional variation. (D) Yearly trends in PCV2 and PCV3 prevalence at both the sample and farm levels, showing a sharp decline after 2022 for PCV2 and relatively stable low endemicity for PCV3. (E) Monthly distribution of PCV2 (E1) and PCV3 (E2) detections, illustrating a pronounced autumn peak for both viruses.

Samples originated from 28 provincial‐level administrative regions across China, revealing marked geographic heterogeneity in viral circulation (Figure 1A). PCV2 positivity was highest in Central China (35.4%, 95% CI:32.6%−38.3%) and Southern China (33.6%,95%CI:30.5%−36.9%), followed by Northern China (31.8%, 95% CI:28.2%−35.5%) (Figure 1B). Although the overall prevalence of PCV3 was lower, relatively higher detection rates were observed in Central China (17.9%, 95% CI:15.4%−20.8%) and Southern China (16.2%, 95% CI:13.9%−18.8%), whereas the lowest prevalence occurred in Northwestern China (8.4%, 95% CI:6.1%−11.3%) and Northeastern China (7.9%, 95% CI:5.7%−10.8%) (Figure 1C). These regional disparities suggest that the distribution patterns of PCV2 and PCV3 may be influenced by climatic conditions, swine population density, and regional immunization strategies.

### 3.2. Temporal and Seasonal Dynamics of PCV2 and PCV3

Both PCV2 and PCV3 showed a clear temporal variation during the surveillance period (Figure 1D). PCV2 prevalence declined sharply from the high levels recorded in 2022 to substantially lower values in 2023–2024, with only a modest rebound in 2025. This pattern may reflect a reduction in viral circulation intensity after the 2022 peak, a change in the composition and intensity of diagnostic submissions over time, or a combination of these factors. PCV3, in contrast, remained at consistently lower prevalence across all years, with only slight decreases after 2022. The limited variation in PCV3 prevalence across the study years suggests a relatively stable endemic pattern within this dataset, in contrast to the more pronounced temporal fluctuations observed for PCV2.

Seasonal analysis further revealed distinct peaks for both viruses in autumn (September–November). PCV2 prevalence reached 35.66% in autumn, significantly higher than that in spring (23.58%) and winter (22.18%). PCV3 showed a similar pattern, with an autumn positivity rate of 18.80%, exceeding that of spring (10.79%) and summer (11.94%). These findings indicate that autumn was the season with the highest observed positivity for both PCV2 and PCV3 in this dataset; however, the unequal duration of seasonal sampling means that this pattern should be interpreted with caution (Figure 1E).

### 3.3. Distribution by Sample Type and Production Stage

**Figure 2:**
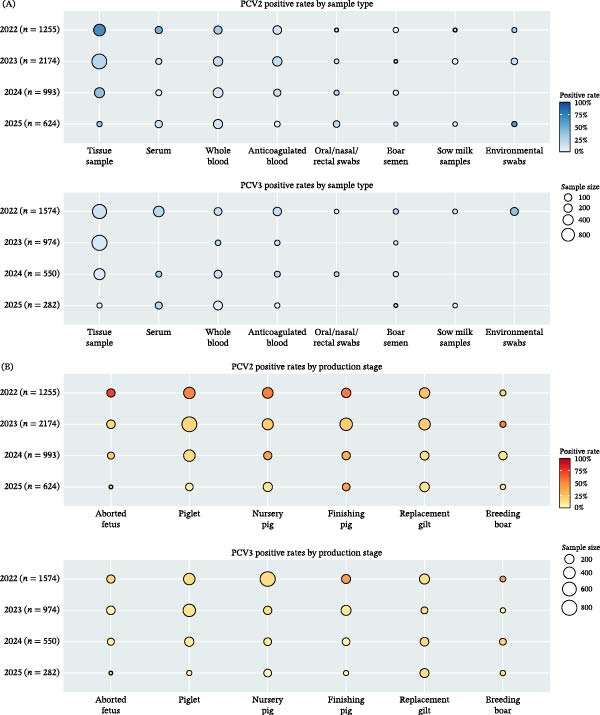
Prevalence of PCV2 and PCV3 across sample types and production stages from 2022 to 2025. (A) Positive rates of PCV2 and PCV3 across sample types. (B) Positive rates of PCV2 and PCV3 across production stages. Bubble size reflects sample size, and color intensity indicates positivity.

Across different sample types, PCV2 was most frequently detected in tissue samples (41.38%, 95%CI: 39.39%−43.38%), followed by swabs (20.43%, 95% CI: 15.09%−26.99%) and serum/plasma samples (16.51%, 95% CI: 13.96%−19.43%). PCV3 showed a distinct pattern, with the highest positivity in swabs (18.18%, 95% CI: 12.96%−24.91%) and serum/plasma (15.28%, 95% CI: 12.92%−17.96%). Notably, PCV3 detection in semen (7.41%) and milk (3.03%) exceeded that of PCV2 in the same sample types, suggesting potential vertical or lactogenic transmission routes for PCV3. However, in this cohort, PCV3 positivity and viral loads in reproductive failure cases were comparable to those in subclinical animals, indicating that its direct contribution to reproductive disorders remains uncertain (Figure 2A).

At the production stage level, PCV2 positivity was highest in aborted fetuses (41.12%, 95% CI: 35.73%−46.71%), followed by finishing pigs (35.24%, 95% CI: 32.06%−38.55%) and nursery pigs (34.65%, 95% CI: 31.59%−37.83%). In contrast, PCV3 infection was more common in boars (22.86%, 95%CI: 15.86%−31.78%) and replacement gilts (16.01%, 95%CI: 13.11%−19.42%). These patterns suggest that PCV2 was frequently detected in samples associated with reproductive failure, which is consistent with its known pathogenic profile. In contrast, PCV3 showed the highest detection rate in boars and replacement gilts, suggesting that this virus may circulate more actively within breeding boars and replacement breeding stock (Figure 2B).

### 3.4. Infection Status and Viral Load by Clinical Category

**Figure 3:**
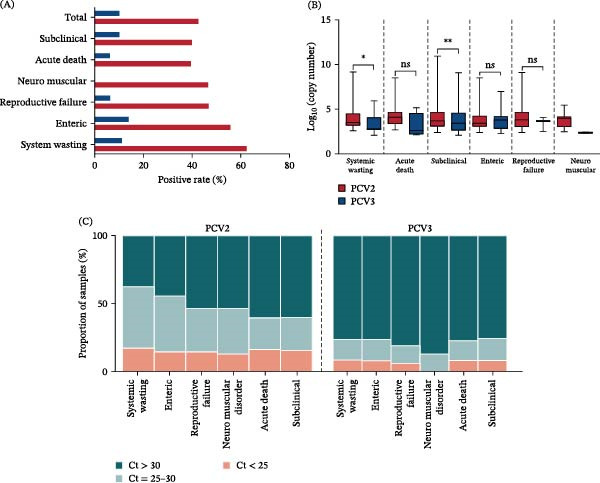
Infection status and viral load profiles of PCV2 and PCV3 across clinical categories. (A) Positive rates of PCV2 and PCV3 among six clinical categories. (B) Viral load distribution of PCV2 and PCV3 across clinical categories. (C) Distribution of Ct value strata for PCV2 and PCV3 across clinical categories.  ^∗^ *p* < 0.05,  ^∗∗^ *p* < 0.01; ns, not significant.

To assess the relationship between clinical presentation and viral infection, 1601 samples with complete background information were categorized into six clinical groups based on field observations: systemic wasting, enteric disorders, reproductive failure, neuromuscular disorders, acute death, and subclinical animals with reduced performance but no overt signs. The overall positivity rates were 42.6% for PCV2 (95% CI: 40.18%−45.04%) and 10.2% for PCV3 (95% CI: 8.80%−11.77%). PCV2 positivity was highest in systemic wasting (62.5%, 95% CI: 56.08%−68.52%) and enteric disorder cases (55.7%, 95% CI: 48.97%−62.20%), whereas PCV3 positivity remained below 15% across all categories (Figure 3A).

Viral load analysis showed that PCV2 copy numbers were significantly higher than those of PCV3 (*p* < 0.001). Among the different clinical categories, PCV2 was more frequently detected in pigs with systemic wasting and enteric symptoms. In contrast, PCV3 viral loads were uniformly low across clinical categories, with no significant differences observed (*p* > 0.05), remaining at overall low levels across groups (Figure 3B). Ct‐based stratification further revealed that PCV2‐positive samples from pigs with systemic wasting and enteric symptoms were more often distributed within the low‐to‐intermediate Ct ranges, whereas the proportion of high Ct samples (Ct > 30) was relatively lower. In contrast, PCV3 samples were predominantly characterized by high Ct values across all clinical categories, suggesting a pattern of low‐level circulation within the pig population (Figure 3C). Collectively, these findings support a stronger association of PCV2 with clinical manifestations than that of PCV3, whereas PCV3 appears more likely to persist widely in pig populations at relatively low viral loads.

### 3.5. Phylogenetic Analysis of PCV2 and PCV3

A total of 181 PCV2 ORF2 sequences and 106 PCV3 ORF2 sequences were obtained for phylogenetic analysis. PCV2 displayed a stable genetic pattern dominated by PCV2d, which accounted for 77.9% (141/181) of all sequences. The remaining genotypes included PCV2a (16.6%, 30/181), PCV2b (5.0%, 9/181), and PCV2g (0.6%, 1/181). (Figure 4A) PCV2d was widely distributed across all major pig‐producing regions, with dense clustering in Northern, Central, and Eastern China, indicating its nationwide spread. By contrast, PCV2a showed a scattered distribution and was mainly detected in Central, Eastern, and Southern China; PCV2b was primarily found in Central and Southern China, whereas PCV2g was identified at only a single site in Southern China. Overall, the spatial distribution of PCV2 was characterized by the broad dissemination of PCV2d, while the other genotypes were detected less frequently and exhibited only localized or sporadic distribution patterns (Figure 4C).

Figure 4Phylogenetic relationships and geographic distribution of PCV2 genotypes and PCV3 ORF2‐based phylogenetic clusters in China. (A) Maximum‐likelihood phylogeny of PCV2 ORF2 sequences. Newly generated sequences from this study are indicated with red stars. The bar plot summarizes the proportional distribution of PCV2 genotypes across sampling years. (B) Maximum‐likelihood phylogeny of PCV3 ORF2 sequences. Newly obtained sequences are marked with red stars. The accompanying bar plot shows the yearly frequencies of PCV3 ORF2‐based phylogenetic clusters. (C) Geographic distribution of PCV2 genotypes. (D) Geographic distribution of PCV3 ORF2‐based phylogenetic clusters.

**Figure:**
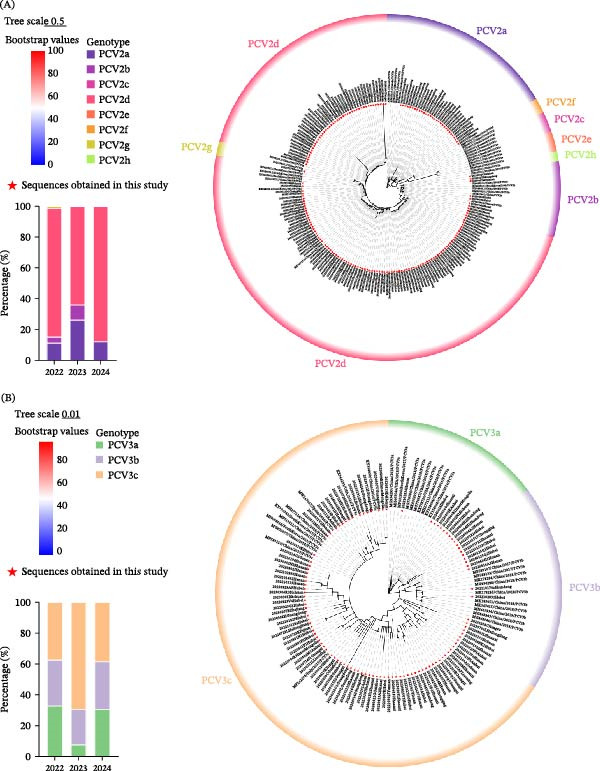

**Figure:**
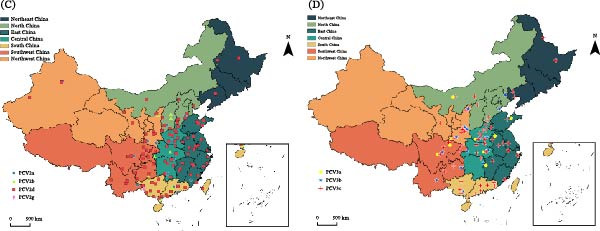

PCV3 exhibited a more diversified ORF2‐based cluster composition but was similarly centered on a single predominant cluster. Among the 106 sequences, PCV3c constituted 45.3%, followed by PCV3b (28.3%) and PCV3a (26.4%) (Figure 4B). PCV3a and PCV3b were mainly detected in Central, Eastern, and Southwestern China, whereas PCV3c predominated in Eastern, Central, and Southern China and was also detected in Northeastern, Northern, Southwestern, and Northwestern China, indicating a relatively broad geographic distribution. Overall, PCV3 showed a nationwide co‐circulation pattern involving multiple ORF2‐based phylogenetic clusters, with differences in detection density among regions and a certain degree of spatial heterogeneity in cluster distribution (Figure 4D).

The persistent dominance of PCV2d indicates that this genotype currently drives the PCV2 circulation in China. PCV3c likewise predominates within the PCV3 population and circulates at a low overall diversity. These patterns highlight the need for ongoing molecular monitoring, especially in breeding herds where covert transmission may occur.

### 3.6. Selection Pressure Analysis of PCV2 and PCV3 ORF2 Genes

Selection pressure analyses integrating four codon‐based methods (MEME, FEL, FUBAR, and SLAC) showed that the ORF2 genes of both PCV2 and PCV3 are predominantly shaped by purifying selection, with overall dN/dS ratios well below 1. This indicates strong evolutionary constraints on the structural integrity of the Cap protein. Despite this genome‐wide purifying pressure, multiple sites under episodic or pervasive positive selection were detected in both viruses (Table 3).

**Table 3:** Summary of positively selected sites in the PCV2 and PCV3 ORF2 gene. (MEME/FEL/SLAC: *p* ≤ 0.1; FUBAR: posterior ≥ 0.9).

Gene	Method	Number of positively selected sites	Positively selected sites (codon position; *p*‐value or posterior probability)
PCV2 ORF2 gene	MEME	25	10 (0.0018), 11 (0.0001), 12 (0.0274), 31 (0.0784), 53 (0.0061), 54 (0.0002), 56 (0.0825), 59 (0.0123), 61 (0.0084), 62 (0.0545), 69 (0.0105), 71 (0.0953), 72 (0.0938), 74 (0.0938), 78 (0.0721), 99 (0.0379), 111 (0.0130), 120 (0.0008), 140 (0.0092), 141 (0.0977), 173 (0.0537), 182 (0.0065), 183 (0.0038), 186 (0.0224), 221 (0.0000)
	FEL	5	8 (0.0880), 53 (0.0650), 120 (0.0847), 141 (0.0960), 173 (0.0455)
	FUBAR	3	3 (0.9075), 8 (0.9429), 46 (0.9152)
	SLAC	0	—
PCV3 ORF2 gene	MEME	15	4 (0.0004), 6 (0.0349), 7 (0.0000), 16 (0.0642), 17 (0.0026), 28 (0.0085), 31 (0.0310), 154 (0.0530), 205 (0.0000), 206 (0.0000), 210 (0.0012), 212 (0.0552), 216 (0.0005), 222 (0.0718), 224 (0.0389)
	FEL	6	4 (0.0095), 16 (0.0290), 28 (0.0020), 31 (0.0109), 154 (0.0229), 210 (0.0005)
	FUBAR	6	4 (0.9667), 16 (0.9827), 28 (0.9859), 31 (0.9833), 154 (0.9669), 210 (0.9964)
	SLAC	3	6 (0.044), 28 (0.039), 210 (0.024)

**Figure 5:**
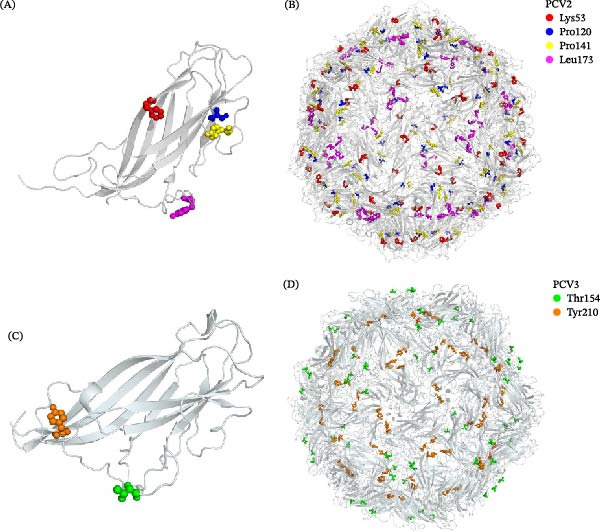
Mapping of positively selected sites on the capsid protein structures of PCV2 and PCV3. (A) PCV2 Cap protein monomer. (B) PCV2 capsid structure showing the distribution of positively selected residues. (C) PCV3 Cap protein monomer. (D) PCV3 capsid structure showing the distribution of positively selected residues. Positively selected residues are shown as colored spheres. The mapped sites in PCV2 were Lys53 (red), Pro120 (blue), Pro141 (yellow), and Leu173 (magenta), whereas those in PCV3 were Thr154 (green) and Tyr210 (orange).

PCV2 exhibited a greater number of positively selected sites than PCV3. Among these, residues Phe8, Lys53, Pro120, Pro141, and Leu173 were consistently identified by multiple methods, representing the most robust selection hotspots (Figure 5A). Two additional sites (Ala3 and Val46) were detected exclusively by FUBAR, suggesting weaker signals of pervasive selection that may reflect minor adaptive adjustments in the PCV2 Cap protein. Combined with structural mapping, the key positively selected sites of PCV2 were mainly distributed in surface‐exposed regions of the Cap protein. In structural mapping, the spatial positions of Lys53, Pro120, Pro141, and Leu173 were clearly visualized in both the monomeric structure and the 60‐mer capsid assembly (Figure 5B), suggesting that host immune selection pressure may play an important role in the accumulation of nonsynonymous variation.

In contrast, PCV3 showed fewer selected sites, with residues Arg4, Arg16, Leu28, Arg31, Thr154, and Tyr210 repeatedly detected across analyses, suggesting potential roles in viral adaptation. Structural mapping further indicated that these sites were mainly located in the N‐terminal and surface‐related regions of the Cap protein. In particular, the spatial positions of Thr154 and Tyr210 were more clearly resolved in both the monomeric structure and the 60‐mer capsid assembly (Figure 5C,D). Overall, although PCV3 exhibits lower overall genetic variation than PCV2, its ORF2 gene still shows limited adaptive change signals.

### 3.7. Phylogenetic and Phylodynamic Analyses Based on the Global Dataset

To reduce the potential temporal structure bias associated with using only Chinese sequences and to better reflect the long‐term evolutionary background of the viruses, this study integrated reference sequences from multiple countries with the sequences generated in this study and constructed datasets comprising 259 PCV2 ORF2 sequences and 147 PCV3 ORF2 sequences with sampling‐time information. The PCV2 dataset included sequences from China, Russia, the United States, India, and Brazil, whereas the PCV3 dataset included sequences from China, South Korea, the United States, Italy, Germany, and Sweden (Supporting Information 2: Table S3 and Supporting Information 3: Table S4).

**Figure 6:**
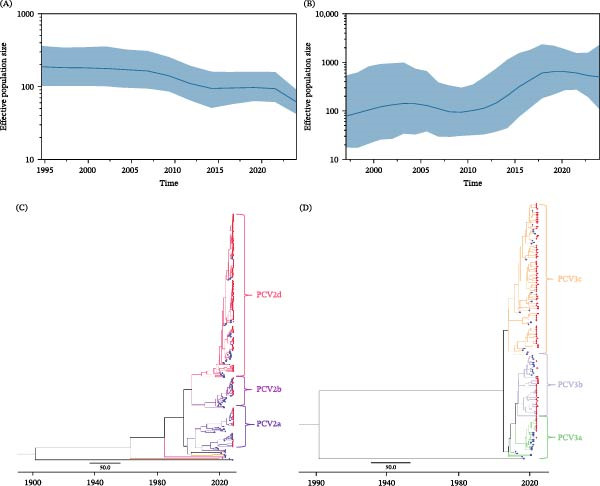
Phylodynamic reconstruction and time‐scaled phylogenetic trees. (A) PCV2 skygrid reconstruction. (B) PCV3 skygrid reconstruction. The solid line represents the median estimate, and shaded areas denote the 95% highest posterior density (HPD) intervals. (C) Time‐scaled maximum clade credibility (MCC) tree of PCV2 inferred under a relaxed molecular clock. (D) Time‐scaled maximum clade credibility (MCC) tree of PCV3 inferred under a relaxed molecular clock. In the MCC trees, red dots indicate sequences obtained in this study, and blue dots indicate reference sequences.

Bayesian time‐scaled analysis estimated the time to the most recent common ancestor (tMRCA) of PCV2 at 1906.54 (95% HPD: 1863.96–1946.25). Coalescent analyses showed that the effective population size (Ne) of PCV2 remained overall at the 10^2^ order of magnitude throughout the study period and exhibited a slow declining trend, with no clear evidence of sustained recent expansion. Its evolutionary rate was estimated at 8.5595 × 10^−4^ substitutions/site/year (95% HPD: 6.4308 × 10^−4^−1.0532 × 10^−3^), indicating a relatively steady long‐term evolutionary pattern. PCV3, in contrast, had an estimated tMRCA of 1912.22 (95% HPD: 1863.61–1951.93). Its effective population size remained relatively stable during the early period, increased gradually after 2013, reached a comparatively higher level between 2015 and 2020, and then remained at that elevated range thereafter. The evolutionary rate of PCV3 was estimated at 4.6026 × 10^−4^ substitutions/site/year (95% HPD: 3.0107 × 10^−4^−6.4427 × 10^−4^). Although the 95% HPD intervals of the two viruses partially overlapped, PCV2 overall showed a relatively faster molecular evolutionary trend than that of PCV3 (Figure 6).

### 3.8. Discrete Phylogeographic Reconstruction Based on the China Provincial Dataset

**Figure 7:**
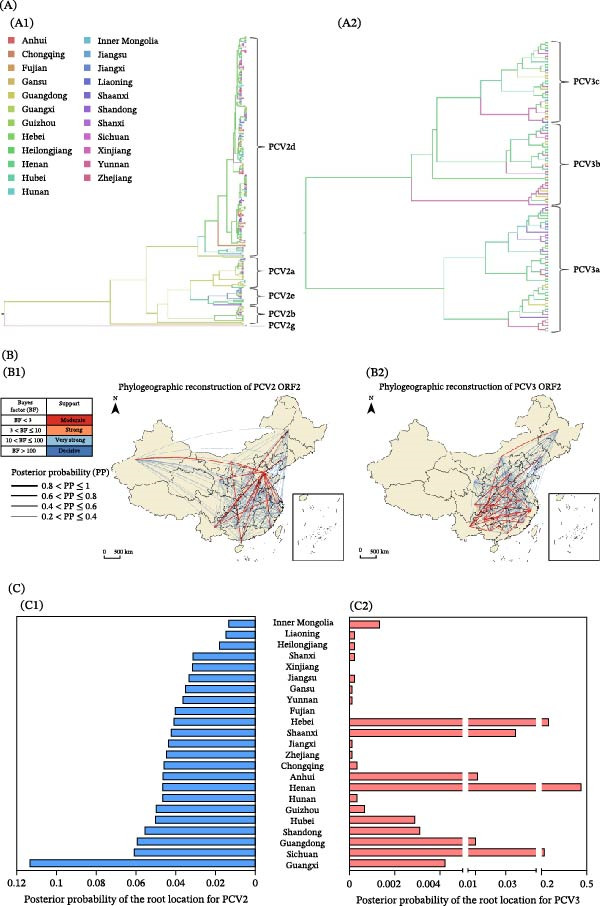
Spatial diffusion patterns and inferred root locations of PCV2 and PCV3 in China. (A) Phylogeographic reconstruction of PCV2 (A1) and PCV3 (A2) based on time‐scaled BEAST analyses. (B) Spatial diffusion networks of PCV2 (B1) and PCV3 (B2) inferred from discrete phylogeographic analysis. (C) Posterior probability distribution of inferred root locations for PCV2 (C1) and PCV3 (C2).

Discrete phylogeographic reconstruction based on the sequences generated in this study showed marked differences between PCV2 and PCV3 in their transmission structures at the provincial level, as illustrated in Figure 7. PCV2 exhibited clear regional clustering in the phylogenetic topology. Some lineages were obviously aggregated within specific regions, suggesting a strongly spatially structured transmission process (Figure 7A). Spatial diffusion analysis further showed a centralized pattern. Hebei acted as a major transmission hub, and multiple statistically supported migration routes were extended to neighboring provinces. Together, these results indicate a directional dissemination pattern radiating outward from the local core regions (Figure 7B,C). In contrast, PCV3 displayed a more admixed phylogeographic structure. Sequences from different regions were interspersed, and geographic clustering was limited. The posterior probabilities of internal nodes were also more dispersed, suggesting more frequent interregional mixing and repeated introductions from multiple sources (Figure 7A,C). Its diffusion pattern involved several potential regional sources, including Guizhou, Sichuan, Fujian, and Henan. However, the inferred transmission routes were more scattered overall, and cross‐regional spread appeared to occur more frequently. This pattern is consistent with a multicentered and weakly structured diffusion process (Figure 7B C).

Overall, PCV2 tended to show clearer regional clustering and more directionally structured dissemination. By contrast, PCV3 exhibited a multicentered and weakly structured diffusion process, which is more consistent with cryptic and dispersed spread under continuous introductions from multiple sources. These findings reflect fundamental differences in the transmission dynamics of the two viruses.

## 4. Discussion

This study provides a comprehensive characterization of the epidemiology, genetic diversity, evolutionary dynamics, and spatial dissemination patterns of PCV2 and PCV3 in China during 2022–2025. Given China’s central role in global swine production and animal movement, these findings provide important reference data for understanding circovirus transmission risks and for guiding regionally and internationally relevant surveillance strategies. Overall, PCV2 exhibited substantially higher prevalence than PCV3 across production stages and clinical categories, consistent with previous reports demonstrating the sustained endemicity and strong adaptive capacity of PCV2 in Asian swine populations [37, 38, 40]. Both viruses showed distinct seasonal peaks in autumn, which may be associated with climatic fluctuations, immune stress, and increased activity of respiratory or enteric pathogens during this period [41].

Because our dataset is based on passive diagnostic submissions rather than structured longitudinal surveillance and detailed information on herd‐level vaccination strategies, African swine fever‐related culling, and other management changes was not available, this temporal pattern should be interpreted cautiously and may partly reflect shifts in case mix or sampling intensity rather than a uniform nationwide reduction in PCV2 circulation.

The notably higher detection of PCV2 in tissue samples, compared with blood‐derived samples, reflects its characteristic tropism toward lymphoid‐ and immune‐associated tissues. By comparison, the detection of PCV3 in semen and milk supports the possibility of vertical or lactogenic transmission, in line with previous reports [42, 43]. Clinically, PCV2 was more frequently detected in pigs with systemic wasting and enteric conditions. PCV2‐positive samples from these categories were more often distributed within the low‐to‐intermediate Ct ranges. This suggests a stronger association between PCV2 and clinical manifestations under field conditions. This observation is consistent with its established role in porcine circovirus‐associated systemic disease and PRDC [38, 44].

PCV3 showed a different pattern. Viral loads remained low across clinical categories, with no significant variation among the syndromes. Most PCV3‐positive samples had relatively high Ct values, suggesting that PCV3 more often circulates at low levels in pig populations than shows a clear association with a specific clinical syndrome. This interpretation is consistent with increasing evidence that PCV3 is frequently detected in clinically healthy or only mildly affected animals. At the same time, its pathogenic significance may still depend on the host status, coinfection, and management conditions [43, 45]. These findings indicate that PCV2 remains the principal target for diagnostic and surveillance efforts. Although PCV3 showed no prominent association with particular clinical signs in this study, it may still persist at low levels and circulate unnoticed within herds. Therefore, continued monitoring of PCV3 in breeding herds remains necessary. These findings highlight the value of large‐scale surveillance in China for understanding the contemporary epidemiology of both circoviruses in intensive pig production systems.

Evolutionary analyses further demonstrated that PCV2d has largely replaced PCV2a and PCV2b as the dominant genotype in China, mirroring national and global trends reported over the past decade [37, 38, 40, 46]. The sustained predominance of PCV2d likely reflects enhanced viral fitness and potential immune escape capacity, raising concerns about reduced cross‐protection from traditional PCV2a‐derived vaccines in some herds. Although PCV3 exhibits lower overall genetic diversity, the co‐circulation of PCV3a, PCV3b, and PCV3c indicates ongoing regional differentiation, consistent with phylogenetic patterns observed in China and internationally [43, 45]. These findings underscore the need to update PCV2 immunization strategies to ensure adequate coverage of PCV2d epitopes and to maintain vigilant molecular surveillance for emerging PCV3 lineages, particularly in breeding and nucleus herds.

Bayesian phylodynamic analyses based on a relaxed molecular clock and coalescent Skygrid models provided additional insights into the long‐term population dynamics of both viruses. To reduce the potential temporal structure bias associated with using only Chinese sequences and to better reflect the long‐term evolutionary background of the viruses, we incorporated reference sequences from multiple countries into the phylodynamic framework. Specifically, the PCV2 dataset included reference sequences from Russia, the United States, India, and Brazil, whereas the PCV3 dataset included reference sequences from South Korea, the United States, Italy, Germany, and Sweden.

For PCV2, Bayesian time‐scaled analysis estimated the tMRCA at 1906.54 (95% HPD: 1863.96–1946.25). The effective population size (Ne) remained overall at the 10^2^ order of magnitude throughout the study period and showed a slow declining trend, with no clear evidence of sustained recent expansion. Its evolutionary rate was estimated at 8.5595 × 10^−4^ substitutions/site/year, which falls within the range previously reported for PCV2 in global datasets [34, 36]. This pattern is compatible with long‐term endemic circulation under relatively stable host and management conditions without evidence of explosive expansion.

For PCV3, the estimated tMRCA was 1912.22 (95% HPD: 1863.61–1951.93). Its effective population size remained relatively stable during the early period, increased gradually after 2013, reached a comparatively higher level between 2015 and 2020, and then remained within that elevated range thereafter. The evolutionary rate of PCV3 was estimated at 4.6026 × 10^−4^ substitutions/site/year. Although lower than some earlier estimates reported for PCV3 [24, 47], this result may reflect differences in dataset composition, sampling range, and analytical framework, and it suggests that the ORF2 region of PCV3 was relatively more conserved under the present dataset. Together, these results indicate that PCV2 is more consistent with long‐term endemic circulation accompanied by slow evolutionary change, whereas PCV3 showed a relatively later increase in population size, although the overall magnitude of expansion remained limited.

Selection pressure analyses further clarified the differences in adaptive evolution between the two circoviruses. Integrating four codon‐based methods (SLAC, FEL, MEME, and FUBAR), both PCV2 and PCV3 ORF2 genes exhibited dN/dS ratios strongly below 1, indicating that the Cap gene is predominantly constrained by purifying selection, consistent with global analyses [24, 47, 48]. Despite this, PCV2 presented a larger number of positively selected sites, with residues 8, 53, 120, 141, and 173 robustly supported across methods. These residues map to exposed surface regions corresponding to reported immunodominant epitopes and putative receptor binding interfaces [36, 49, 50], suggesting localized adaptive evolution under host immune pressure and possibly vaccine‐related selection. PCV3, by contrast, had fewer and weaker positively selected sites, although its adaptive codons were also clustered within exposed structural segments. This pattern is consistent with a largely conserved genome with only limited local adaptation [43, 48, 51]. Overall, PCV2 appears more strongly shaped by immune‐driven selection, whereas PCV3 variation is more closely linked to cryptic transmission dynamics and ecological heterogeneity among host populations.

Phylodynamic and phylogeographic reconstructions further revealed fundamental differences between PCV2 and PCV3 in their transmission structures. PCV2 displayed a well‐supported, geographically structured phylogeny with high posterior probabilities (≥0.85) and distinct regional clustering, particularly in Hebei, Shandong, and Henan. Hebei acted as a major dissemination hub, and several strongly supported migration pathways extended to Jiangsu, Inner Mongolia, Chongqing, Xinjiang, Hunan, and Sichuan, forming multidirectional diffusion patterns from north to south and from east to west, which is consistent with large‐scale swine movement flows within core production regions [21, 46]. This centralized structure suggests that targeted surveillance and control efforts in key hub regions may disproportionately reduce downstream transmission to surrounding provinces, highlighting the importance of region‐specific biosecurity and movement management strategies.

PCV3 showed a more admixed phylogeographic pattern, with sequences from different regions interspersed across the time‐scaled tree and with multiple independent diffusion sources. Guizhou, Sichuan, Fujian, and Henan constituted key transmission nodes, forming slow but persistent multidirectional spread networks. This decentralized pattern aligns with the subclinical persistence of PCV3 and its tendency for multipoint introduction facilitated by animal movement across breeding and production systems [43, 48, 51]. Such cryptic and multisource transmission may allow PCV3 to circulate unnoticed within breeding and replacement herds, complicating early detection and underscoring the need for sustained molecular surveillance even in the absence of overt clinical disease. An alternative explanation is that PCV3 may be equally endemic but more difficult to track due to lower viral loads, historically lower sequencing intensity, and underreporting, so the apparently weaker spatial structure and more recent expansion could partly reflect detection and sampling biases rather than fundamentally different movement networks.

Several limitations of this study should be acknowledged when interpreting the phylodynamic and phylogeographic inferences. First, although the BEAST analyses were based on the ORF2 region rather than complete genomes, this is likely a relatively minor limitation because ORF2 is one of the most widely used and phylogenetically informative genomic regions in circovirus research. It can provide a relatively stable basis for inferring major genetic groupings, temporal structure, and overall evolutionary trends while also reducing analytical bias associated with recombination. Therefore, ORF2 is well suited for investigating the broad evolutionary patterns addressed in this study. Second, although international reference sequences were incorporated into the phylogenetic datasets used for time‐scaled and phylodynamic analyses, publicly available sequences from outside China remained limited. Their temporal and geographic distribution was also uneven, particularly for PCV3 in earlier years and in some regions.

In addition, the discrete phylogeographic reconstruction in this study was specifically designed to examine the transmission structure among provinces within China. Accordingly, geographic states were defined at the provincial scale within China rather than at the country level, and the inferred diffusion pathways should therefore be interpreted as reflecting relative patterns of domestic spread rather than a full reconstruction of the international transmission history. Although introductions from outside China may have contributed to the current genetic diversity of both viruses, especially during the early emergence and spread of PCV3, the currently available historical sequences from outside China remain limited and unevenly distributed across regions and years. Under these conditions, combining international locations with provincial locations in a single discrete phylogeographic framework could introduce additional biases because of differences in geographic scale and sampling density.

In this study, ORF2 was selected as the primary analytical target for both PCV2 and PCV3. ORF2 encodes the major capsid protein and represents the most immunologically relevant and evolutionarily informative genomic regions of circoviruses. In addition, ORF2 remains the most widely sequenced region in large‐scale surveillance studies, particularly for retrospective and multiregional datasets, enabling robust temporal, phylogenetic, and phylogeographic comparisons across different studies and regions. The ORF2 sequences analyzed in this study were generated by Sanger sequencing of PCR amplicons, which produces consensus sequences dominated by the major variant present within each host. This approach is appropriate for defining the prevailing lineage in each sample but does not capture low‐frequency variants or minor co‐circulating lineages, and dN/dS estimates based on such consensus data are therefore likely to underestimate the true standing genetic variation and the intensity of positive selection acting within hosts. As a result, the inferred expansion of PCV3 Ne and the apparent differences in spatial structure between PCV2 and PCV3 should be considered hypothesis‐generating rather than definitive reconstructions of their long‐term evolutionary histories. Future studies incorporating broader international sampling, more balanced temporal coverage, and preferably complete genome data will be needed to more rigorously assess the contribution of cross‐border introductions to the epidemiological patterns observed in China.

In conclusion, this study highlights clear contrasts between PCV2 and PCV3 in their epidemiology, evolutionary dynamics, and spatial transmission patterns within China. PCV2 remains the dominant pathogenic circovirus, characterized by centralized diffusion and strong associations with clinical diseases, suggesting that targeted control in key transmission hubs may be particularly effective. In contrast, PCV3 circulates at lower viral loads and shows a geographically admixed and cryptic transmission pattern, posing challenges for detection and long‐term control. Together, these findings emphasize the importance of integrating molecular surveillance with spatial and evolutionary analyses to inform region‐specific and transboundary disease monitoring strategies in intensive swine production systems.

## Author Contributions

Qigai He, Xuexiang Yu, and Wentao Li conceived the project and supervised the overall study. Yanhong Chen and Qigai He designed the study. Yanhong Chen, Yi Lu, Yang Zeng, Ling Dong, and Mingkai Lei performed the bench experiments and data analysis. Yanhong Chen wrote the first draft of the manuscript. Yanhong Chen, Yaofang Hu, Panpan Li, Weidong Yan, Anan Jongkaewwattana, Wentao Li, Xuexiang Yu, and Qigai He critically revised and edited the manuscript.

## Funding

This work was supported by the National Natural Science Foundation of China (Grant 32302866) and the China Agriculture Research System of MOF and MARA (Grant CARS‐35).

## Disclosure

All authors read and approved the final manuscript.

## Conflicts of Interest

The authors declare no conflicts of interest.

## Supporting Information

Additional supporting information can be found online in the Supporting Information section.

## Supporting information


**Supporting Information 1** Table S1 Information of PCV2 reference strains. Tables S2 Information of PCV3 reference strains.

**Supporting Information 2** Table S3 Information on PCV2 ORF2 sequences used for global phylogenetic and phylodynamic analyses.

**Supporting Information 3** Table S4 Information on PCV3 ORF2 sequences used for global phylogenetic and phylodynamic analyses.

## Data Availability

The data that support the findings of this study are available from the corresponding author upon reasonable request.
