# Supplementary material for: Molecular Surveillance and Evolutionary Dynamics of Porcine Circovirus Types 2 and 3 in China, 2022–2025
Source: Transbound Emerg Dis. 2026 Jun 9;2026:6670465. doi: 10.1155/tbed/6670465 (PMC13247986; doi:10.1155/tbed/6670465)
Supplement: Supplementary file 1 — Supporting Information 1 Table S1 Information of PCV2 reference strains. Tables S2 Information of PCV3 reference strains. [file TBED-2026-6670465-s001.docx]

**Supplementary Tables 1** Information of PCV2 reference strains.

| Accession | Genotype | Origin | Year |
| --- | --- | --- | --- |
| AF027217.1 | PCV2a | / | / |
| AF055392.1 | PCV2a | Canada | / |
| HQ202948.1 | PCV2a | Taiwan | / |
| KT868159.1 | PCV2a | USA | 2006 |
| JQ806749 | PCV2a | / | / |
| KP975444.1 | PCV2a | / | / |
| KX960947.1 | PCV2a | / | / |
| EU386606.1 | PCV2a | Sweden | 1993 |
| AF055394.1 | PCV2b | France | / |
| AY686764.1 | PCV2b | China | / |
| FJ598044.1 | PCV2b | China | 2008 |
| FJ644919.1 | PCV2b | China | 2002 |
| HM038018.1 | PCV2b | China | 2005 |
| KC620533.1 | PCV2b | South Korea | 1999 |
| EU148503.1 | PCV2c | Denmark | / |
| EU148504.1 | PCV2c | Denmark | / |
| EU148505.1 | PCV2c | Denmark | / |
| KJ094599.1 | PCV2c | Brazil | 2010 |
| KX161675.1 | PCV2d | China | 2007 |
| KX828234.1 | PCV2d | South Korea | 2016 |
| KX960917.1 | PCV2d | China | 2002 |
| KC620553.1 | PCV2d | South Korea | 2003 |
| KC620554.1 | PCV2d | South Korea | 2003 |
| KC620555.1 | PCV2d | South Korea | 2005 |
| KT867797.1 | PCV2e | USA | 2006 |
| KT867798.1 | PCV2e | USA | 2012 |
| KT867799.1 | PCV2e | USA | 2006 |
| KT867800.1 | PCV2e | USA | 2012 |
| MF278777.1 | PCV2f | China | 1999 |
| LC008137.1 | PCV2f | India | / |
| HM776452.1 | PCV2f | China | 2009 |
| DQ363860.1 | PCV2g | China | / |

**Supplementary Tables 2** Information of PCV3 reference strains.

| Accession | Genotype | Origin | Year |
| --- | --- | --- | --- |
| MG934295 | PCV3a | China | 2015 |
| KY354054 | PCV3a | China | 2016 |
| MF631804 | PCV3a | South Korea | 2016 |
| KY354068 | PCV3a | China | 2016 |
| MH277111 | PCV3a | China | 2018 |
| MG770384 | PCV3a | China | / |
| MG770387 | PCV3a | China | / |
| KX778720 | PCV3a | USA | 2015 |
| MK033207 | PCV3a | China | 2017 |
| MH603546 | PCV3a | USA | 2017 |
| MF589107 | PCV3b | China | 2017 |
| MF589133 | PCV3b | China | 2017 |
| MK178295 | PCV3b | China | 2018 |
| MK185651 | PCV3b | China | 2017 |
| MK347415 | PCV3b | China | 2016 |
| MG934298 | PCV3b | China | 2016 |
| MK178284 | PCV3b | China | 2018 |
| MN605934 | PCV3b | China | 2018 |
| MG868943 | PCV3b | China | 2017 |
| MG868940 | PCV3b | China | 2017 |
| KX966193 | PCV3c | USA | 2016 |
| KY354050 | PCV3c | China | 2016 |
| MF162298 | PCV3c | Italy | 2017 |
| MW167067 | PCV3c | Spain | 2020 |
| KY996343 | PCV3c | South Korea | 2016 |
| MN698814 | PCV3c | South Korea | 2016 |
| MH277114 | PCV3c | China | 2018 |
| MF769811 | PCV3c | China | 2017 |
| MF589110 | PCV3c | China | 2016 |
| MG870097 | PCV3c | China | 2018 |
